# Supplementary material for: ATP and NAD+ Deficiency in Parkinson’s Disease
Source: Nutrients. 2023 Feb 14;15(4):943. doi: 10.3390/nu15040943 (PMC9961646; doi:10.3390/nu15040943)
Supplement: Supplementary file 1 [file nutrients-15-00943-s001.zip › Figure S2.pdf]

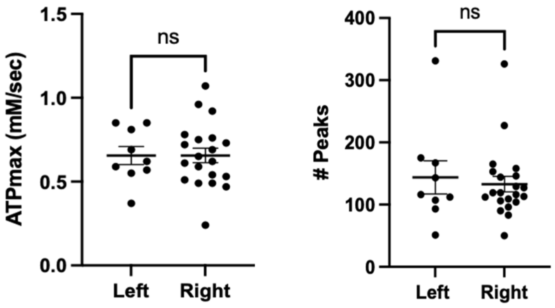

Figure S2: Comparing effect of side of onset of PD symptoms

Comparison of skeletal muscle phenotypes between subjects with predominantly left or right sides affected by PD. (A) TA ATPmax; (B) TA muscle endurance (number of peaks).
