# Supplementary material for: ATP and NAD+ Deficiency in Parkinson’s Disease
Source: Nutrients. 2023 Feb 14;15(4):943. doi: 10.3390/nu15040943 (PMC9961646; doi:10.3390/nu15040943)
Supplement: Supplementary file 1 [file nutrients-15-00943-s001.zip › Table S1.pdf]

Table S1: List of inclusion and exclusion criteria for PD subjects
**Inclusion Criteria**
• Age 65-85 years.<br>• Ability to read and speak English<br>• Ability to attend a 3-hour study visit in Seattle, WA<br>• Hoehn & Yahr Stage 2-3 (bilateral disease, not severely disabled).
**Exclusion Criteria**
• Any Contra-indication to MRI (pacemaker, electronic implant, claustrophobia, pregnancy etc.)<br>• History of epilepsy, stroke, brain surgery, or structural brain disease.<br>• The presence of other serious illnesses (e.g. concurrent cancer treatment)<br>• Current or recent enrollment in a clinical trial involving an investigational product or device, or a medical research (causing incompatibility with the study).<br>• Any supplementation in the previous 30 days, including NAD, nicotinamide mononucleotide (NMN), nicotinamide riboside (NR), and other nutraceuticals designed to target NAD.<br>• Current drug or alcohol use or dependence.<br>• Inability/unwillingness to provide informed consent (e.g. diagnosis of dementia, confusion about study goals or participation).<br>• Acute infection (e.g. upper respiratory, dermal) in the previous 30 days.<br>• Limb tremor or dyskinesia that cannot be comfortably controlled for 90 minutes.
