# Supplementary material for: ATP and NAD+ Deficiency in Parkinson’s Disease
Source: Nutrients. 2023 Feb 14;15(4):943. doi: 10.3390/nu15040943 (PMC9961646; doi:10.3390/nu15040943)
Supplement: Supplementary file 1 [file nutrients-15-00943-s001.zip › Table S2.pdf]

Table S2: PD symptom severity correlations with Leg [NAD], Leg [ATP] and Hand [ATP] by simple linear regressions. Non-normally distributed variables were square root transformed.

	Leg [NAD]	Leg [ATP]	Hand [ATP]
	β±SE	β±SE	β±SE
UPDRS pt1	-0.012±0.02	-0.008±0.08	-0.0057±0.03
UPDRS pt2	0.018±0.01	-0.009±0.006	-0.012±0.01
UPDRS pt3	0.008±0.008	**-0.01±0.005***	**-0.016±0.006***
UPDRS pt4	0.014±0.02	-0.014±0.01	-0.029±0.02
UPDRS total	0.0058±0.004	-0.004±0.003	**-0.0079±0.004***
√PRO-PD total	-0.0005±0.008	-0.008±0.004	0.0019±0.007
√PRO-PD non-motor	-0.004±0.01	-0.008±0.006	0.0012±0.009
PRO-PD motor	0.0004±0.0005	**-0.0006±0.0002***	0.00005±0.0004
√Slow	0.017±0.02	**-0.003±0.002***	0.0068±0.02
√Constipation	0.026±0.02	**-0.029±0.01***	-0.028±0.02
√Walking	-0.023±0.02	**-0.028±0.01***	0.011±0.02
√Freezing	0.01±0.02	-0.027±0.01	-0.011±0.02
√Falling+	0.028±0.02	**-0.03±0.01****	-0.013±0.02
√Rising+	0.040±0.03	-0.013±0.01	-0.016±0.02
√Daily living	0.033±0.02	-0.010±0.01	0.002±0.02
√Motivation+	-0.036±0.02	-0.002±0.01	0.017±0.02
Handwriting	-0.0006±0.002	-0.0003±0.001	0.00068±0.002
√Depression	-0.008±0.02	-0.004±0.01	0.0023±0.02
√Interest+	**-0.04±0.02***	-0.0055±0.01	0.024±0.02
√Anxiety	-0.03±0.02	-0.013±0.01	-0.010±0.02
Fatigue	-0.0045±0.003	-0.0020±0.001	0.0035±0.003
√Daytime Sleepiness	-0.005±0.04	-0.01±0.02	-0.018±0.02
√Dyskinesia+	-0.01±0.02	-0.017±0.01	0.013±0.02
√Tremor+	0.002±0.002	**-0.028±0.01***	-0.018±0.02
√Balance+	0.0032±0.002	**-0.030±0.01***	-0.00066±0.02
√Temp. Dysregulation+	0.019±0.02	-0.016±0.01	-0.0069±0.02
√Orthostasis+	-0.020±0.02	-0.0095±0.01	**0.036±0.01***
Visual	0.0027±0.002	**-0.026±0.01***	-0.0016±0.002
√Insomnia+	0.020±0.02	0.0033±0.01	-0.017±0.02
REM sleep behavior	**-0.04±0.02***	-0.012±0.01	0.0034±0.002
√Dystonia	0.0046±0.02	-0.0036±0.01	0.016±0.02
√Speech+	-0.0057±0.02	0.0060±0.01	0.0033±0.02
√Drool	-0.0017±0.02	-0.018±0.01	-0.033±0.02
√Stoop	0.013±0.03	-0.029±0.02	-0.0035±0.02
√Memory	0.00046±0.02	-0.021±0.02	-0.01±0.02
√Comprehension	-0.017±0.02	-0.016±0.01	0.0031±0.02
√Smell+	-0.01±0.02	-0.011±0.01	-0.015±0.014
√Medication Side Effects+	-0.012±0.02	-0.0075±0.01	-0.0017±0.02
√Sexual Dysfunction+	0.008±0.02	0.0052±0.008	-0.016±0.01
√Urinary Symptoms	0.046±0.02	0.0066±0.01	0.016±0.02
√Hallucinations+	-0.013±0.02	-0.0079±0.02	-0.019±0.02
√Nausea+	-0.010±0.02	**-0.042±0.02****	-0.034±0.02
PROMIS Score	0.0017±0.01	-0.00058±0.006	0.0042±0.008

UPDRS = Unified Parkinson's Disease Rating Scale; PRO-PD = Patient Reported Outcomes in Parkinson's Disease; PROMIS = Patient reported outcome measurement information system.
+Remained non-normally distributed after transformation
*p=0.05
**p=0.01
